# Molecular MR Imaging of Prostate Cancer

**DOI:** 10.3390/biomedicines9010001

**Published:** 2020-12-22

**Authors:** Avan Kader, Julia Brangsch, Jan O. Kaufmann, Jing Zhao, Dilyana B. Mangarova, Jana Moeckel, Lisa C. Adams, Ingolf Sack, Matthias Taupitz, Bernd Hamm, Marcus R. Makowski

**Affiliations:** 1 Department of Radiology, Charité—Universitätsmedizin Berlin, Corporate Member of Freie Universität Berlin, Humboldt-Universität zu Berlin, and Berlin Institute of Health, Charitéplatz 1, 10117 Berlin, Germany; julia.brangsch@charite.de (J.B.); jan-ole.kaufmann@charite.de (J.O.K.); jing.zhao@charite.de (J.Z.); dilyana.mangarova@charite.de (D.B.M.); jana.moeckel@charite.de (J.M.); lisa.adams@charite.de (L.C.A.); ingolf.sack@charite.de (I.S.); matthias.taupitz@charite.de (M.T.); bernd.hamm@charite.de (B.H.); marcus.makowski@tum.de (M.R.M.); 2 Department of Biology, Chemistry and Pharmacy, Institute of Biology, Freie Universität Berlin, Königin-Luise-Str. 1-3, 14195 Berlin, Germany; 3 Department of Veterinary Medicine, Institute of Animal Welfare, Animal Behavior and Laboratory Animal Science, Freie Universität Berlin, Königsweg 67, Building 21, 14163 Berlin, Germany; 4 Division 1.5 Protein Analysis, Federal Institute for Materials Research and Testing (BAM), Richard-Willstätter-Str. 11, 12489 Berlin, Germany; 5 Department of Chemistry, Humboldt-Universität zu Berlin, Brook-Taylor-Str. 2, 12489 Berlin, Germany; 6 Department of Veterinary Medicine, Institute of Veterinary Pathology, Freie Universität Berlin, Robert-von-Ostertag-Str. 15, Building 12, 14163 Berlin, Germany; 7 School of Biomedical Engineering and Imaging Sciences, King’s College London, St Thomas’ Hospital Westminster Bridge Road, London SE1 7EH, UK; 8 Department of Diagnostic and Interventional Radiology, School of Medicine & Klinikum Rechts der Isar, Technical University of Munich, Munich (TUM), Ismaninger Str. 22, 81675 München, Germany

**Keywords:** prostate cancer, magnetic resonance imaging, molecular imaging, molecular marker

## Abstract

This review summarizes recent developments regarding molecular imaging markers for magnetic resonance imaging (MRI) of prostate cancer (PCa). Currently, the clinical standard includes MR imaging using unspecific gadolinium-based contrast agents. Specific molecular probes for the diagnosis of PCa could improve the molecular characterization of the tumor in a non-invasive examination. Furthermore, molecular probes could enable targeted therapies to suppress tumor growth or reduce the tumor size.

## 1. Introduction

Prostate cancer (PCa) is one of the most frequent cancers diagnosed in men and the fifth leading cause of death worldwide [1]. In 2018, 1,276,106 new PCa diagnoses were registered globally [1]. PCa is a malignant neoplasia of the prostate gland in men. The etiology of prostate cancer remains still unknown, but there are certain well-established risk factors that affect the development and progression of the disease. These factors include environmental factors such as diet and living conditions, but also genetic components [2,3,4,5]. The most important risk factor is advanced age [3]. The mortality rate is strongly age-dependent and increases from the age of >65 [1].

In the early stages of the disease, patients can be symptom-free. If symptoms like bone pain, urinary dysfunction, weight loss and anaemia occur, metastases have usually already formed, especially in the local lymph nodes or skeleton [6]. The majority of patients are diagnosed with intermediate or terminal PCa, which poses major challenges for treatment and recovery. Fortunately, diagnosis and treatment of PCa have improved in recent years due to medical progress. Screening for elevated blood plasma levels of prostate-specific antigen (PSA) at an early stage is considered an effective approach to the early detection and immediate treatment of PCa [2,7].

The current clinical reference standard is rectal palpation of the prostate. If changes in the size or consistency of the prostate are detected, a blood sample is taken to determine the patient’s PSA level. This is followed by a digital rectal examination (DRE) and/or a transrectal ultrasound examination (TRUS) [8]. A punch biopsy is performed to analyse the tissue. If a malignant disease of the prostate tissue is detected, the spread of the tumor in the body is determined by means of conventional magnetic resonance imaging (MRI) and/or computer tomography (CT). Other imaging techniques used to diagnose local PCa include multiparametric MRI (mpMRI), positron emission tomography (PET) or an MRI examination followed by a punch biopsy. mpMRI relies on several parameters, such as T1- and T2-relaxation, diffusion-weighted imaging and perfusion imaging. During PET examination, a positron emitter and a tracer are used to image specific metabolic processes for which radiopharmaceuticals are administered. In an MRI examination, a contrast agent is often used to show differences in the tissue structure.

Molecular MRI is currently an intensely researched field. Molecular imaging enables the visualization of biological processes in vivo at the molecular level. Cells are subject to constant reorganization, are in constant exchanges with neighbouring cells and there are continuous changes in biochemical processes. In this context, molecular imaging offers a specific insight into the living organism without surgical intervention. A specific biomarker conjugated to a paramagnetic element is used for this purpose and causes a signal change in MRI. Molecular imaging uses specific probes that target on biological ligands, like prostate-specific membrane proteins and receptor molecules. The target can be conjugated with MR active metal, like Gd^3+^ or Fe^3+^. Based on the lock-and-key principle, the probes bind to the target and enrichment follows. Detection is realized with imaging techniques. This approach can be crucial for the diagnosis, early detection and treatment of PCa.

The incidence and mortality rates of PCa remain high. Further research is urgently needed to clarify the onset and mechanism of PCa progression [3]. This article focuses on the diagnosis of PCa using molecular MR imaging.

## 2. Pathophysiology of Prostate Carcinoma

The origin of PCa still remains largely unknown. Several processes are involved in the development of PCa: The precursor intraepithelial neoplasia followed by localized prostate cancer and advanced prostate adenocarcinoma [9]. Androgens play an important role in the development of PCa [10]. While it was previously believed that the development of PCa was associated with high testosterone levels [11], recent research has shown that testosterone is not responsible for the development of PCa, but has a promoting effect in the development of the tumor [12,13,14,15].

Prostate gland tissue consists of three main types of epithelial cell types: Luminal, basal and neuroendocrine [16,17]. This morphological division is also found in mice, which facilitates the development of PCa mouse models. Luminal epithelial cells are located in the prostate lumen and express a high concentration of androgens (AR) [18]. They are positive for CD57 (epitope for the antibody human natural killer-1 (HNK-1) [19]), NKX3.1 (androgen-regulated homedomain gene; expression is predominantly localized to prostate epithelium [20]), prostate-specific antigen (PSA) and cytokeratins 8 and 18 (epithelium-specific intermediate-sized filament with 19 subtypes [21]) [18,22]. Basal epithelial cells separate the lumen from the stroma, express less AR and are positive for cytokeratins 5 and 14, p63 (prostate basal cell marker of some PCa subtypes [23]), CD44 (a cell-surface protein involved in glucose metabolism of prostate cancer cells [24]) and GSTP1 (belongs to glutathione S-transferases family; enzymes active in detoxification of exogenous substances; involved in the regulation of the cell cycle [25]) [18]. Luminal and basal cells are considered to be the cellular origin of prostate cancer [26] and play an essential role in PCa progression [18].

Histone H3-variant-centromere-protein A (CENPA), KIF20A and CDCA8 are hub genes that are overexpressed in PCa development and tumor progression [27]. CENPA is an epigenetic marker that is highly overexpressed in prostate cancer tissue and cell lines [28]. The PCa stage correlates with the CENPA level [28]. In the study by Saha et al., data from sequencing experiments indicate that CENPA serves as a transcriptional regulator for the expression of proliferation genes, cell cycle genes and centromere genes and thus plays a decisive role in the development of prostate tumors [28]. KIF20A is a kinesin-like protein and plays a role in mitosis (important factor in the chromosome passenger complex) [29]. An increased expression of KIF20A has been found in the development of various tumor diseases [30,31,32]. Zhang et al. analysed the effect of KIF20A in PCa on proliferation, migration and invasion of PCa cells [29]. Patient data as well as in vitro and in vivo experiments were used. The analysis of patient data showed that a high expression resulted in a lower chance of survival [29]. When the expression of KIF20A was downregulated in vitro and in vivo, it suppressed proliferation, migration and invasion of PCa cells. Further studies are needed to determine the therapeutic potential.

Stroma cells (fibroblasts, macrophages, lymphocytes, mast cells, endothelial cells, pericytes and smooth muscle cells) and extracellular matrix proteins play an essential role in tumor initiation and progression [33,34,35]. Fibroblasts are specific connective tissue cells and are an important component in the formation and degradation of the extracellular matrix (ECM) [36]. In the presence of a tumor they can transform into cancer-associated fibroblasts. This cell type is particularly common in the prostate [37]. Macrophages are cells of the innate immune system and are responsible for tissue homeostasis [38]. Tumor-associated macrophages are found in prostate cancer and play a crucial role in the infiltration of inflammatory cells. Stroma cells can promote tumor growth due to their complex intracellular communication [39]. Studies have shown that tumor-associated macrophages of type M1 have an inhibitory effect on tumor cells, as opposed to type M2, which have a promoting effect on the growth of the tumor [39]. This finding was also confirmed with PCa [40]. Another type of cell that plays an important role in the development of PCa is lymphocytes, a subtype of leucocytes. Patients with PCa show a lower level of lymphocytes [41]. A study by Adhyatma et al. suggested that the neutrophil-lymphocyte ratio could also be used to determine PCa before a punch biopsy [42]. In tumors, mast cells act on growth and influence angiogenesis [43,44]. Tumor subtypes must be further explored in order to possibly generate mast cells as biomarkers [45,46]. Current research shows many different opinions about the function and influence of mast cells on the tumor [46]. Further research is needed to determine the exact function of mast cells in PCa. Endothelial cells play a role in the neovascularization of PCa and, as biomarkers, could provide good information on the biochemistry of PCa and monitor the effectiveness of treatment [47]. A study by Zhao et al. focused on the role of endothelial cells in prostate cancer progression and found in cell culture experiments that the cells secrete C-C motif chemokine ligand 5 and induce autophagy by suppressing AR expression [48]. Inhibition of this mechanism could inhibit metastasis [48]. Pericytes are involved in the development and maintenance of vascular systems. They regulate vascular permeability, vascular wall stability and are involved in the regulation of blood flow, but also in the interaction for vascular compression and maturation [49]. During tumor development/tumor angiogenesis, pericytes migrate into heterogenic tumor tissue, are freely bound to the endothelium and unorganized [49]. The extracellular matrix consists of collagen, fibronectin, tenascin, versican, galectin, laminin, elastin and others [50]. These proteins are important for cell adhesion and cell signaling. They influence the restructuring of the ECM, which can have a positive effect on tumor architecture [50]. In the formation of PCa, it is not only the tumor cells that are crucial, but also the microenvironment of the tumor.

The interaction between different cells is essential for the progression of cancer. At the same time, knowledge about the components could be used to develop biomarkers that help to detect PCa at an early stage and use them as a tool in the therapy and treatment process.

## 3. Clinical Diagnosis

When diagnosing PCa, a classification called TNM (T= volume of tumor; N= involvement of lymph nodes; M= metastasis classification) is carried out first (see Table 1) [51]. The diagnosis of prostate cancer is usually based on transrectal ultrasound (TRUS) evaluation, often combined with a needle biopsy to obtain up to twelve tissue samples for histological examination. While biopsy remains the diagnostic standard, there are some limitations to this technique. A biopsy represents only a small section of the total tissue [52]. It is also invasive for the patient and can lead to bleeding, inflammation and infection. Another serious problem may arise if tumor cells seed into surrounding tissue, such as perirectal or rectal tissue, during the prostate biopsy [53,54].

Prostate-specific antigen (PSA), a tumor marker, can be used to diagnose PCa stage. A high PSA level may indicate a malignant tumor of the prostate, inflammation of the prostate or prostate hyperplasia. It is observed that diagnosis is increasingly made at an earlier stage of PCa. With a PSA level between 2.5 ng/mL and 4 ng/mL, PCa is diagnosed in about 30% of biopsies [55]. This means that in 7 out of 10 patients a prostate biopsy is performed, which is not necessary. In an ultrasound-guided punch biopsy, tissue is punched out of several regions of the prostate and examined histologically. On the contrary, a case report shows that PSA may not be expressed in all patients with PCa [56].

To better identify the stage of the tumor, MR imaging techniques are used to assist in the standard examination. An endorectal coil with a phased array coil of medium to high field strength in the region of the pelvis is used for an MRI examination [57]. T2-weighted images (3 mm transverse, sagittal and coronal sequences) are acquired for detection and staging PCa [58]. A transverse T1-weighted sequence is performed to detect possible intraglandular bleeding [58]. The pelvic phased-array coil increases the signal-to-noise ratio during imaging to improve resolution [58]. mpMRI is increasingly used for the detection of PCa. Prostate tumors, which develop in the transitional zone, can be detected by T2-weighted imaging. T2-weighted imaging, diffusions-weighted imaging and dynamic contrast-enhanced MRI are used to analyse the tissue architecture, sensitivity and specificity of PCa. The images are interpreted differently in interpreting of these data, so the Prostate Imaging Reporting and Data System (PI-RADS) was introduced [59,60]. This aims to standardise the reporting of PCa. So far, the success has been moderate [61]. The newer version leads to better reproducibility of the readers [62]. An improvement could be archieved by other parameters, such as MR proton spectroscop or MR elastography, which represents stiffness and tissue fluidity [63].

However, the detection of PCa depends on many conditions and sometimes shows a lower signal change. This may be due to the subtype of PCa, bleeding, hyperplastic nodes and/ or treatment [58]. To minimize the risk to the patient, to avoid invasive characterization of the tumor by pathological examinations and to ensure reliable detection, biomolecular MRI contrast agents have to be designed that represent the clinical picture of PCa at the molecular level without intervention. To this end, it is necessary to develop target molecules depending on the subtype of prostate cancer.

## 4. Molecular Magnetic Resonance Imaging (MRI)

Magnetic resonance imaging is a non-ionizing imaging modality based on a strong magnetic field and radio waves. Tumors can be easily detected by MRI because they are generated with a high spatial and temporal resolution [64,65,66]. Contrast media containing gadolinium (III) (Gd) dominate diagnostic MRI for many tumorous diseases. Until recently, Magnevist^®^, Omniscan^®^, Dotarem^®^, Gadovist^®^ and Multihance^®^ are the most commonly used contrast media in hospitals [67,68]. Studies using Magnevist^®^ and Omniscan^®^ have shown that Gd is released and deposited in the tissue [69]. Since the beginning of 2018, these two contrast media are no longer authorized to be used. Contrast agents are responsible to increase the longitudinal T1 relaxation rate and the transverse T2 relaxation rate of H_2_O molecules [68].

Contrast media containing Gd have been criticised for some time. Gd has been shown to be deposited in the brain, skin, heart and kidneys, especially in patients with nephrogenic systemic fibrosis (NSF) [70,71]. Studies that investigated the deposition of Gd in humans have been able to detect Gd in biopsies of NSF patients [71,72,73,74,75,76]. Linear contrast media based on Gd were found to release free Gd^3+^ ions in the body due to their unstable structure [69,77]. In addition, a distinction is made between ionic and non-ionic contrast media. Ionicity is related to osmolarity. This factor (linear or macrocyclil; ionic or non-ionic) plays a role in the tolerance of contrast agent for patient/ organism [78].

As the administration of contrast agent can be indispensable, it is important to pay attention to how often a patient receives a dose and whether he or she has any health restrictions. A better alternative would be to use a contrast agent with a stable structure, which allows no or little free Gd to enter the organism and be deposited. A relatively new method to achieve this is molecular imaging, which makes it possible to visualize biological structures or metabolic processes in cells.

Cells in an organism are constantly changing [79,80,81,82,83], both before the onset of disease, during a disease and during its course [81]. Tumor diseases are a good example: tumor cells use and absorb nutrients differently from healthy tissue [84,85]. Because of the altered metabolic processes, it is possible to determine the change in the organism by using specific markers in the region of interest [86]. Molecular imaging aims to visualize in vivo characterization at the cellular and molecular level using special contrast agents [87]. This allows for an assessment of the molecular nature of the tumor without the need for a biopsy.

### 4.1. Peptide-Based Contrast Medium

Peptide-based molecular imaging enables specific statements to be made about the molecular nature of a disease. Conventional contrast agents used so far have a low level of targeting or non-targeting. By contrast, targeted molecular imaging can be used for early diagnosis, detection and monitoring the treatment process. Disease related changes in the metabolism of an organism can be targeted with probes. For some time now, probes that bind to peptide receptors, for example, have been continuously developed to achieve a high binding affinity [88]. Overexpressed molecules and receptors are ideal to act as biomarkers [89]. In molecular imaging, it is necessary to develop targeted molecular contrast agents that have a high specificity in prostate tumor tissue and are not toxic to the individual.

A major advantage of using peptide-based contrast agent is their small size and the paramagnetic Gd^3+^ compound, which causes a signal increase in T1-weighted images. Therefore, it is possible to dock to more receptors compared to antibody-based preparations [90]. CA1.CD2 (ProCA1) has a strong metal binding affinity and metal-binding selectivity and may have a good reflexivity [88]. Wei et al. further developed the contrast agent. A gastrin release sequence was added, which allows better targeting [91]. This gastrin-release peptide (GRP) binds specifically to the surface receptors GRPR, which are frequently found in large numbers in prostate cancer [91]. This contrast agent is modified to target the surface receptor with the use of ten amino acid peptide from the *C*-terminus of GRP [91]. The binding affinity is due to the loop structure of the *C*-terminus, which explains the better targeting, specifically in prostate cancer/prostate tumors [91]. The experiments were performed in different PCa tumor cell lines and additionally a tumor xenograft mouse model was used for the in vivo studies. For MRI examination, 140 µM of contrast agent was injected into the tumor tissue directly [91].

**Figure 1:**
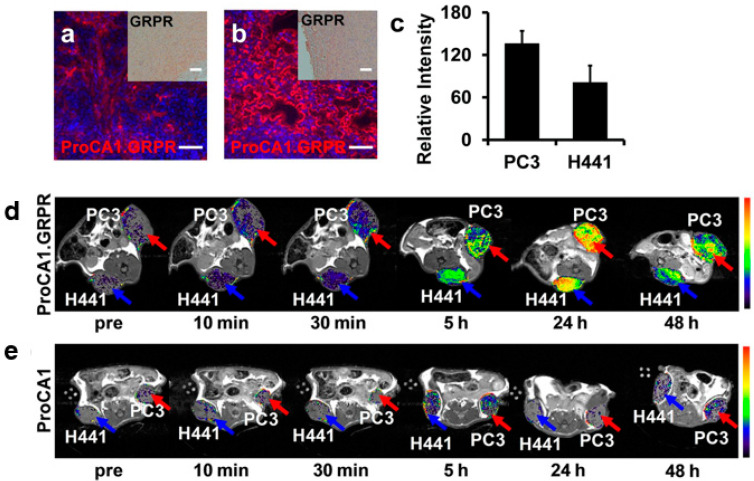
ProCA1.GRPR distribution and gastrin-releasing receptor (GRPR) expression in xenografted tumor tissue from Pu et al. 2015 (modified); (**a**,**b**) Immunofluorescence staining of ProCA1.GRPR targeting GRPR on (**a**) H441 and (**b**) PC3 tumors in xenograft mice. Blue: nucleus staining. Red: Staining GRPR on (**a**) H441 and (**b**) PC3 tumor. (**c**) Graphical representation of fluorescence intensity of ProCA1.GRPR staining in PC3 and H441 tumors. PC3 tumor shows a stronger expression as H441 tumors. (**d**) T1-weighted spin echo MR imaging of ProCA1.GRPR targeting GRPR in H441 (blue arrow) and PC3 (red arrow) in mice (xenograft). (**e**) T1-weighted spin echo MR imaging of non-targeted ProCA1 in H441 (blue arrow) and PC3 (red arrow) in mice (xenograft). Scale bar = 100 μm. Colorbars show the signal intensity (from high (red) to low (black)).

Pu et al. focused on the development of a gastrin-releasing receptor (GRPR)-directed MRI contrast agent with a specific Gd^3+^ binding site (PRoCA1.GRPR), which was detected by MRI in tumors of mice [92]. ProCA1.GRPR shows a high reflaxivity and a strong binding affinity for GRPR to physiological metal ions [92]. Studies suggest that gastrin-releasing peptides promote tumor growth [93,94]. Figure 1a,b show the ProCA1.GRPR distribution and GRPR expression in the explanted tumor tissue from two different mouse PCa cell lines, PC3 and H441. The corresponding MRI images (Figure 1d,e) illustrate the specific binding of the contrast agent by a higher signal intensity after contrast agent administration by tail vein injection, with an eight times lower clinical injection dose (5 mM) [92].

GRPR is proving to be a good indicator for prostate cancer and is a promising target for molecular imaging. It could be used in the early detection of cancer as well as in the treatment monitoring of such patients. In this context, it would be important to analyse the different tumors at the molecular level as far as possible, since different intensities of GRPR are already shown in the two cell lines. GRPR is not only overexpressed in PCa, but also in breast cancer, pancreatic cancer, colorectal cancer and lung cancer [94].

In another study by Pu et al. (2016), a protein MRI contrast agent (ProCA) was further developed to target prostate-specific membrane antigen (PSMA) (ProCA32.PSMA) [95]. The contrast agent shows high Gd^3+^ binding affinity and metal selectivity, relaxivity and a strong PSMA targeting [95]. The experiments were examined on xenograft tumor mice model with a dose of 5 mM of contrast agent. PSMA is an ideal target because it is expressed about ten times more in PCa than in healthy tissue and it is not expressed in other cancers [95].

**Figure 2:**
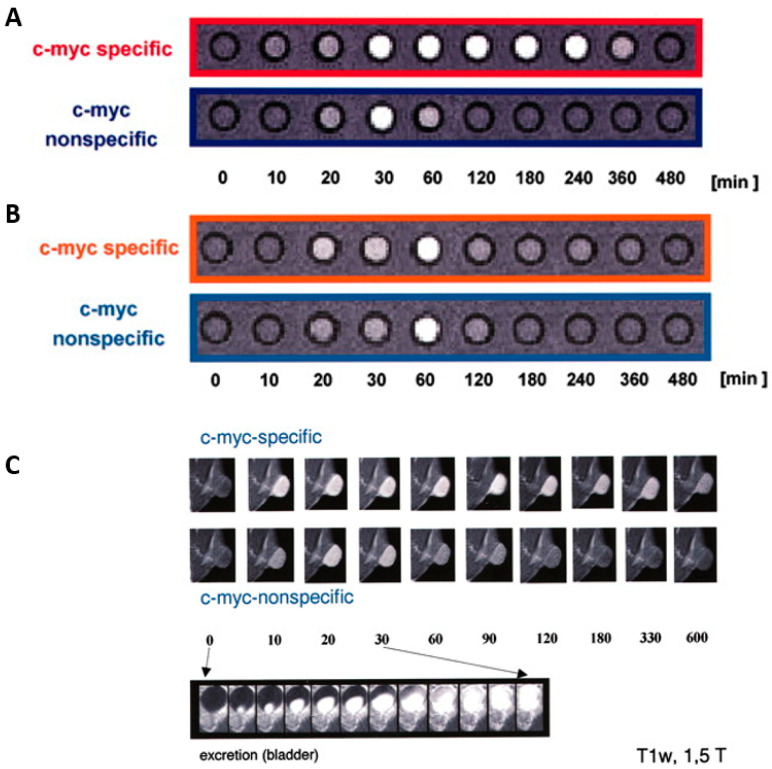
MR signal intensity versus time with c-*myc*-specific Gd^3+^ complex from Heckl et al. 2003 (modified); (**A**) (red) The signalintensity of the c-*myc*-Gd^3+^ comlex for HeLa cells after incubation, and (**B**) by lymphocytes. These are axial T1 weighted images of the cell pellets, in Minimum Essential Media (MEM), each consisting of 20 × 10^6^ cells/ lymphocytes. (**C**) The intensity after intravenous injection of AT-1 (Dunning R- 3327) rat with prostate adenocarcinoma by coronal T1-weighted MR images (TR: 600 ms/TE: 15 ms; scan time, 45 s). All experiments were performed using a 1.5 Tesla Siemens Magnetom.

Heckl et al. developed an intravital and intracellular MRI contrast agent consisting of peptide nucleic acid and a transmembrane carrier peptide coupled to a Gd-complex, c-*mys*-specific Gd^3+^ complex [96]. In the MRI, HeLa cells showed a signal increase after 10 min and reached its maximum after 1 h (Figure 2A). A 1.5 Tesla Siemens Magnetom MRI scanner was used with acquisition of T1- weighted images. A non-specific contrast agent (Magnevist^®^) was administered for comparison. The relaxivity after incubation with the specific contrast agent increased by more than a factor of three compared to the non-specific contrast agent. The experiment was also performed on lymphocytes (Figure 2B). Finally, the experiment was performed in a rat model (AT-1 (Dunning R- 3327)). The contrast agent was administered intravenously to the rat (0.25 μmol/kg) and reached its maximum after 30 min (Figure 2C). A high expression of c-*myc* mRNA was confirmed in the cytoplasm of AT1 (Dunning R- 3327) rat prostate primary tumor and HeLa cells [96]. Another experiment in the study shows that healthy cells (e.g., lymphocytes, brain, liver, lung, muscle and spleen cells) show low expression of c-*myc* messenger ribonucleic acid (mRNA). Further studies are needed to investigate if this contrast agent can also be used for other tumors. In addition, toxicity tests must be performed to determine whether the contrast agent causes cell damage.

**Figure 3:**
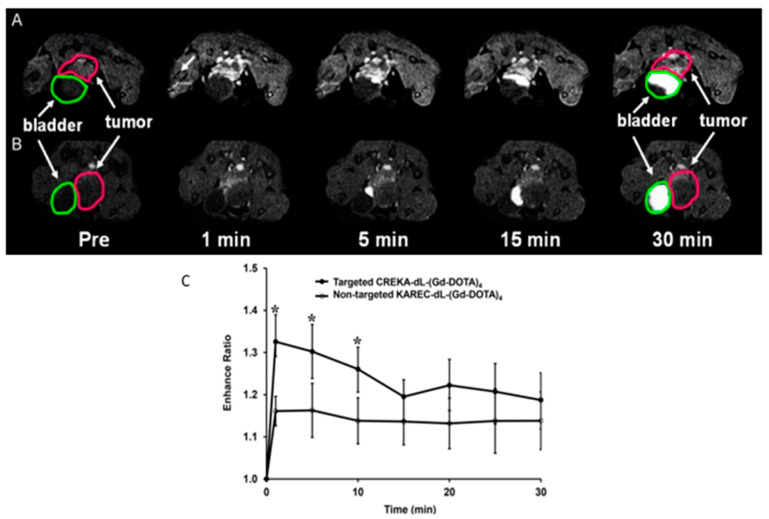
Illustration of signal amplification of CREKA-dL-(Gd-DOTA)_4_ from Wu et al. 2014 (modified); (**A**) T1-weighted axial 2D images of orthotopic prostate cancer mice model before and at different time points after injection of CREKA-dL-(Gd-DOTA)_4_. (**B**) T1-weighted axial 2D images of orthotopic prostate cancer mice model before and at different time points after injection of KAREC-dL-(Gd-DOTA)_4_. The animals were injected with 0.03 mmol-Gd/kg in nu/nu nude mice. (**C**) Signal amplification ratio in tumor tissue: The ratio of signal intensity after contrast agent administration to that before contrast agent administration, up to 30 min after contrast agent injection, * *p* < 0.05.

Fibronectin is strongly expressed in aggressive PCa and is a feature of the epithelial-mesenchymal transition. A contrast agent targeting a fibrin-fibronectin complex was synthesized by Wu et al. (CREKA-dL-(DOTA-Gd)_4_) [97]. The contrast agent was prepared by solid-phase peptide synthesis. The contrast agent was investigated in an orthotopic mouse model. 5 min after administration of the contrast agent (0.03 mmol-Gd/kg), a strong signal amplification was observed for at least 30 min, measured with a Bruker Biospec 7 Tesla MRI scanner with a volume radiofrequency coil (Figure 3A). A non-specific contrast agent was used for comparison (KAREC-dL-(DOTA-Gd)_4_) with the same dose (Figure 3B). The contrast agent shows high water solubility, high relaxivity, small size, tumor specificity and strong signal enhancement [97]. Despite the good results, further studies are necessary to investigate the contrast agent extensively. For example, it must be ruled out that tissue damage can occur if the contrast agent is administered once or several times.

In a study by Tan et al. a contrast agent was modified to target the fibrin-fibronectin complex in the tumor stroma [98]. It is further developing a contrast agent based on the cyclic peptide CLT1 (CGIIQKNEC), which contains the nanoglobular Gd-DOTA monoamide conjugate CLT1-G2-(Gd-DOTA) with a smaller size [98]. Due to its smaller size, it can diffuse more easily into tumor tissue. The specific binding of the CLT1 peptide was investigated with a dose of 30 μmol-Gd/kg and confirmed in the orthotopic tumor mouse model [98]. Despite the good results, further preclinical studies will have to follow, possibly in another animal model to show the target-specific binding in PCa.

In aggressive tumors, the protein extradomain B fibronectin (EDB-FN) is expressed [99]. Four peptides, GVK, IGK, SGV and ZD2, were identified by Li et al. and used for molecular imaging as peptide-GD-DOTA conjugates [100]. The in vivo experiments were examined in the mouse model, by administering 0.1 mmol/kg of respective peptide-GD-DOTA contrast media [100]. The contrast agents show good relaxivity and high water solubility [100]. In contrast to the control (ProHance^®^, Gadoteridol) a better tumor image could be achieved in MRI examination [100]. EDB-FN specific contrast media could become an important component in the identification of aggressive tumors, which also represents a limitation.

### 4.2. Iron Oxide Nanoparticle

Superparamagnetic iron oxide nanoparticles (SPION) are magnetic iron particles with a size of 1-100 nm. SPIONs are used as clinical contrast agents for MRI examinations for diagnosis or treatment of tumors. For the most part, SPION contrast agents are considered negative contrast agents. The accumulation of ferrous contrast agent shows a hypointensive contrast in MRI. After injection of the contrast agent into the bloodstream, the particles are phagocytized by macrophages [101]. In contrast to Gd-containing contrast agents, the relaxation time of T1- and T2-weighted images is shortened by ferrous contrast agents [102]. In recent years, interest in the development of novel SPIONs has increased.

Another specific marker is the prostate-specific membrane antigen (PSMA) on the cell surface. Currently, PSMA is the most widely used biomarker for PCa [95,103]. PSMA is a membrane-bound glycoprotein that consists of 750 amino acids in the human individual. It is a 100 kDa glutamate carboxypeptidase II [95]. PSMA is found in both healthy prostate cells and prostate cancer cells. It plays an important role in nutrient uptake, receptor function, signal transduction and cell migration [95]. PSMA is more strongly expressed by a tumor in the prostate, but also by the downregulation of androgen receptors. Several studies have shown that PSA is associated with PSMA [95,104,105,106]. Patients with elevated PSA levels also show high expression of PSMA. Good results have often been achieved in the treatment of tumor cells that express PSMA [95].

**Figure 4:**
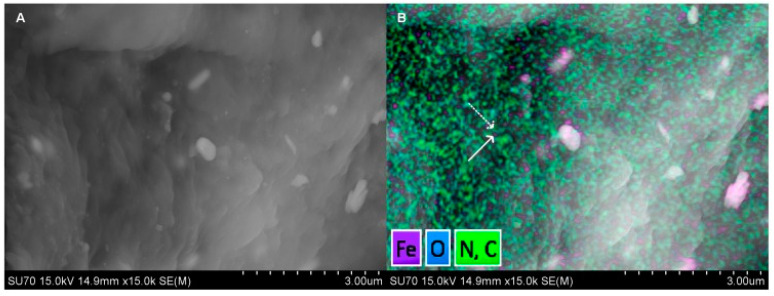
Molecular characterization of muJ591:MIRK from Bates et al. (2014); (**A**) a Scanning Electron Micrograph (SEM) image. (**B**) A superposition of the elements C, O, N and Fe (SEM mapping with Energy Dispersive X-ray (EDX)). Soild arrow shows the iron signal (MIRB nanoparticles) and the dashed arrow shows the antibody as organic matter signal. For Scanning Electron Micrograph (SEM) imaging, the sample must be completely dry. The result of this step is the formation of crystalline salts from the buffer (large white structures).

In a study by Bates et al., a commercially available superparamagnetic iron oxide-containing contrast agent (MIRB- Molday ION Rhodamine-B carboxyl) was linked to a deimmunized mouse monoclonal antibody (muJ591): muJ591:MIRB [107]. The target structure is PSMA. Using Inductively Coupled Plasma- Atomic Emisson Spectrometry (ICP-AES), it could be shown that the modified contrast agent had a loading concentration of 1958 ± 611 (n = 8) elemental iron per antibody and was thus stably conjugated. Other detection methods used are flow cytometry and immunofluorescence. The experiments were conducted on cells (Figure 4) [107].

**Figure 5:**
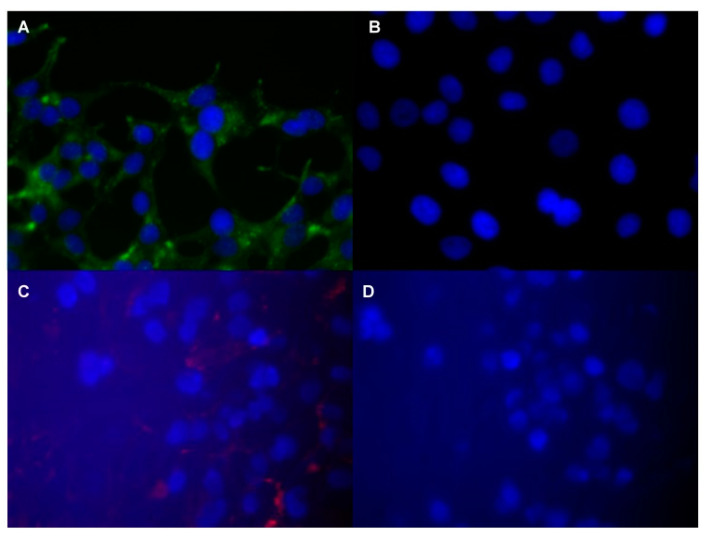
Fluorescence microscopic images of prostate specific tumor cells with muJ591:MIRB from Bates et al. (2014); The images show antibody staining. (**A**) Extracellular and subcellular area LNCaP cells. (**B**) DU145 cells as negative control. (**C**) Rhodamine-B fluorophore detection (red) on LNCaP cells. (**D**) DU145 cells as negative control for detection.

Antibody staining was performed by immunofluorescence microscopy of LNCaP cells expressing PSMA. The secondary antibody conjugated to AlexaFluor-488 was used to bind to rhodamine-B fluorophore (shown in red). MIRB is bound to rhodamine-B fluorophore and could be detected in the LNCaP cells (Figure 5). Figure 5C shows the binding of the newly conjugated contrast agent by means of antibodies. The negative control is DU145 cells that do not express PSMA. No binding of the contrast agent is shown in DUC145 cells.

The results indicate specific binding to PSMA-expressing cells with the modified muJ591:MIRB. However, it is important to investigate the contrast agent in vivo in the future.

The nanopharmaceutical Ferumoxytol (Feraheme^®^) was modified by Kaittanis et al. for use in PCa [108]. In the study, cell experiments (in vitro) were performed and then the nanopharmaceutical was examined in animal models (in vivo) for its therapeutic potential [108].

Ferumoxytol consists of a glucose-based coating that coats an iron oxide. A small cyclic PSMA targeting peptide was conjugated to Ferumoxytol, which allowed the functional state of the androgen receptor pathway to be assessed by MRI and used therapeutically [108].

Another study reports on the PSMA-targeting polypeptide CQKHHNYLC conjugated to SPIONs [109]. In the study by Zhu et al., a polypeptide (CQKHHNYLC)-SPION contrast agent is conjugated, which binds specifically to PSMA [109]. In the in vitro experiment, more polypeptide SPIONs were taken up by the LNCaP cells than by the reference cells [109]. In the tumor-induced mouse model, a significant decrease in T2 tumor signal intensity was observed after injection of the contrast agent (0.240 mg/mL) [109]. Histological examinations confirm the assumption that the signal reduction due to the uptake of the contrast agent was observed in the tumor tissue [109].

### 4.3. New Potential Biomarkers

**Figure 6:**
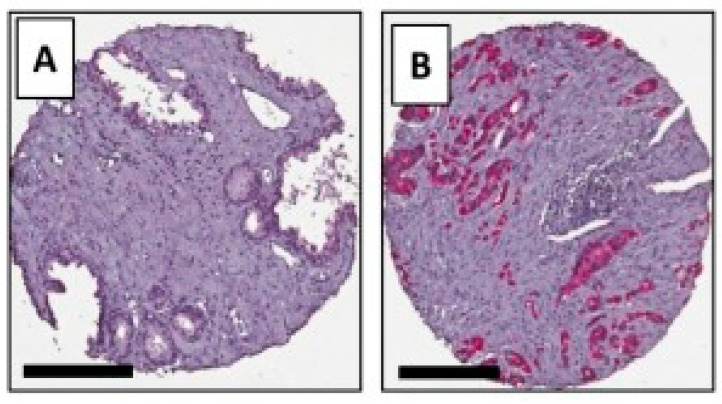
Immunohistological staining of AMACR from Shapovalova M. et al. 2018 (modified); Comparison between (**A**) a healthy prostate and (**B**) prostate cancer. Scale bars = 200 μm.

Alpha-methylacyl-CoA racemase (AMACR) is also a protein that is overexpressed in PCa. It is a mitochondrial and peroxisomal enzyme. AMACR is involved in the metabolism of branched-chain fatty acid and bile acid intermediates [110]. No basal cells were found in the expression of AMACR in PCa by immunohistological investigations of the gland [111]. Shapovalova et al. were able to show that primary and metastatic PCa have higher expression of AMACR than a healthy prostate (Figure 6) [112]. The expression of the AMACR protein was shown and confirmed in LNCaP, PC3 and 22v1 cells [112,113]. In this study, the promoter was used for the detection of the protein and not the protein itself. Another advantage is that the expression of the protein is independent of the androgen receptor (AR) [113]. AR have a direct influence on the transcription of certain genes, which leads to a stronger synthesis of proteins. The knowledge gained could be used to localize primary tumors as well as metastatic tissue using an AMACR-based contrast agent.

One study found that prostate stem cell antigen (PSCA) has an increased expression in prostate tissue [114,115]. Increased PSCA expression indicates the stage of disease and the progression to androgen independence [116]. PSCA is a glycosylphosphatidylinositol-branched membrane protein and plays an important role in microdomain and subcellular signal transduction [117]. PSCA is not only overexpressed in prostate cancer, but also in other types of cancer [118], where it may be involved in tumor progression. By downregulating the gene, it was possible to influence tumor suppression in stomach and bile cancer [117]. It can therefore be used therapeutically. Whether it has the potential to be used as a biomarker is a question of future research.

For visualisation of these target structures with MRI, the specific biomarkers could be labeled with MR active metal complexes, like Gd-DOTA. Afterwards, by ex vivo relaxivity tests, the eligibility of the new probe can be estimated before the probe can be examined in an animal model.

## 5. Conclusions

Molecular MRI contrast agents are a promising tool for the diagnosis and treatment of prostate cancer. Molecular MR imaging offers the potential to detect PCa with high spatial resolution compared to PET. There are several promising approaches for novel contrast agents that need to be further explored before they can eventually be used in patients suffering from PCa.

## Figures and Tables

**Table 1:** Classification of prostate cancer stage (TNM; T= volume of tumor; N= involvement of lymph nodes; M= metastasis classification).

**T**	**Characteristics**		
T1	Clinically asymptotic, non-palpable tumor		
T1a	Histological analysis of prostate tissue with < 5% tumorous tissue		
T1b	Histological analysis of prostate tissue with >5% tumorous tissue		
T1c	Tumor detection by punch biopsy, elevated PSA level		
T2	Palpable tumor, localized in the prostate		
T2a	Maximal half tumor flap tumorous		
T2b	More than half of a prostate lobe is tumorous		
T2c	Both prostate lobes affected		
T3	Volume of tumor over the prostate capsule		
T3a	Tumor spread on one or both sides beyond the prostate capsule, seminal vesicles not affected		
T3b	Extension into the seminal vesicle		
T4	Tumor is fixed and/ or adjacent tissue involved		
**N**	**Characteristics1**	**M**	**Characteristics**
N0	No regional lymph nodes	M0	No metastasis
N1	Regional lymph nodes are existing	M1	Metastasis are existing
NX	Regional lymph nodes cannot be identified	M1a	No regional lymph nodes
		M1b	In bones
		M1c	Other areas
		MX	Metastasis cannot be identified
